# How neurons exploit fractal geometry to optimize their network connectivity

**DOI:** 10.1038/s41598-021-81421-2

**Published:** 2021-01-27

**Authors:** Julian H. Smith, Conor Rowland, B. Harland, S. Moslehi, R. D. Montgomery, K. Schobert, W. J. Watterson, J. Dalrymple-Alford, R. P. Taylor

**Affiliations:** 1 Physics Department, University of Oregon, Eugene, OR 97403 USA; 2 School of Pharmacy, University of Auckland, Auckland, 1142 New Zealand; 3 School of Psychology, Speech and Hearing, University of Canterbury, Christchurch, 8041 New Zealand; 4 New Zealand Brain Research Institute, Christchurch, 8011 New Zealand

**Keywords:** Computational neuroscience, Scientific data, Computational science, Neuroscience

## Abstract

We investigate the degree to which neurons are fractal, the origin of this fractality, and its impact on functionality. By analyzing three-dimensional images of rat neurons, we show the way their dendrites fork and weave through space is unexpectedly important for generating fractal-like behavior well-described by an ‘effective’ fractal dimension *D*. This discovery motivated us to create distorted neuron models by modifying the dendritic patterns, so generating neurons across wide ranges of *D* extending beyond their natural values. By charting the *D*-dependent variations in inter-neuron connectivity along with the associated costs, we propose that their *D* values reflect a network cooperation that optimizes these constraints. We discuss the implications for healthy and pathological neurons, and for connecting neurons to medical implants. Our automated approach also facilitates insights relating form and function, applicable to individual neurons and their networks, providing a crucial tool for addressing massive data collection projects (e.g. connectomes).

## Introduction

Many of nature’s fractal objects benefit from the favorable functionality that results from their pattern repetition at multiple scales^1–3^. Anatomical examples include cardiovascular and respiratory systems^4^ such as the bronchial tree^5^ while examples from natural scenery include coastlines^6^, lightning^7^, rivers^8^, and trees^9,10^. Along with trees, neurons are considered to be a prevalent form of fractal branching behavior^11^. Although previous neuron investigations have quantified the scaling properties of their dendritic branches, typically this was done to categorize neuron morphologies^2,3,12–25^ rather than quantify how neurons benefit from their fractal geometry. Why does the body rely on fractal neurons rather than, for example, the Euclidean wires prevalent in everyday electronics? Neurons form immense networks within the mammalian brain, with individual neurons exploiting up to 60,000 connections in the hippocampus alone^26^. In addition to their connections within the brain, they also connect to the retina’s photoreceptors allowing people to see and connect to the limbs allowing people to move and feel. Given this central role as the body’s ‘wiring’, we focus on the importance of fractal scaling in establishing the connectivity between the neurons^11^. Previous analysis over small parts of the pattern created by a neuron’s dendritic arbor identified scale invariance—the repetition of pattern statistics across size scales—as one of the geometric factors used to balance connectivity with its maintenance costs^27,28^. Their research built on Ramón y Cajal’s wiring economy principle from a century earlier which proposed that neurons minimize their wiring costs. These costs include metabolic expenditures^29,30^, wire volume^31–33^, and signal attenuation and delay^34–36^.

In order to determine the precise role of the scale invariance along with an appropriate parameter for describing it, we first need to address more fundamental questions—to what extent are neurons really fractal and what is the geometric origin of this fractal character? To do this, we construct 3-dimensional models of rat neurons using confocal microscopy (“Methods”). We show that, despite being named after trees, dendrites are considerably different in their scaling behavior. Trees have famously been modeled using a fractal distribution of branch lengths. While dendrites have a range of lengths, the ways in which they fork and weave through space are also important for determining their fractal character. We demonstrate that fractal dimension *D* is a highly appropriate parameter for quantifying the dendritic patterns because it incorporates dendritic length, forking, and weaving in a holistic manner that directly reflects the neuron’s fractal-like geometry. Serving as a measure of the ratio of fine to coarse scale dendritic patterns, we use *D* to directly map competing functional constraints—the costs associated with building and operating the neuron’s branches along with their ability to reach out and connect to other neurons in the network. By investigating ~1600 distorted neuron models with modified dendrite length, forking, and weaving behavior, we propose that the neuron *D* values reflect a network cooperation that optimizes these constraints, with connectivity outweighing cost for neurons with high *D* values. Remarkably, *D* captures this functional optimization even though the fractal-like scaling behavior occurs over a highly limited range of size scales.

**Figure 1:**
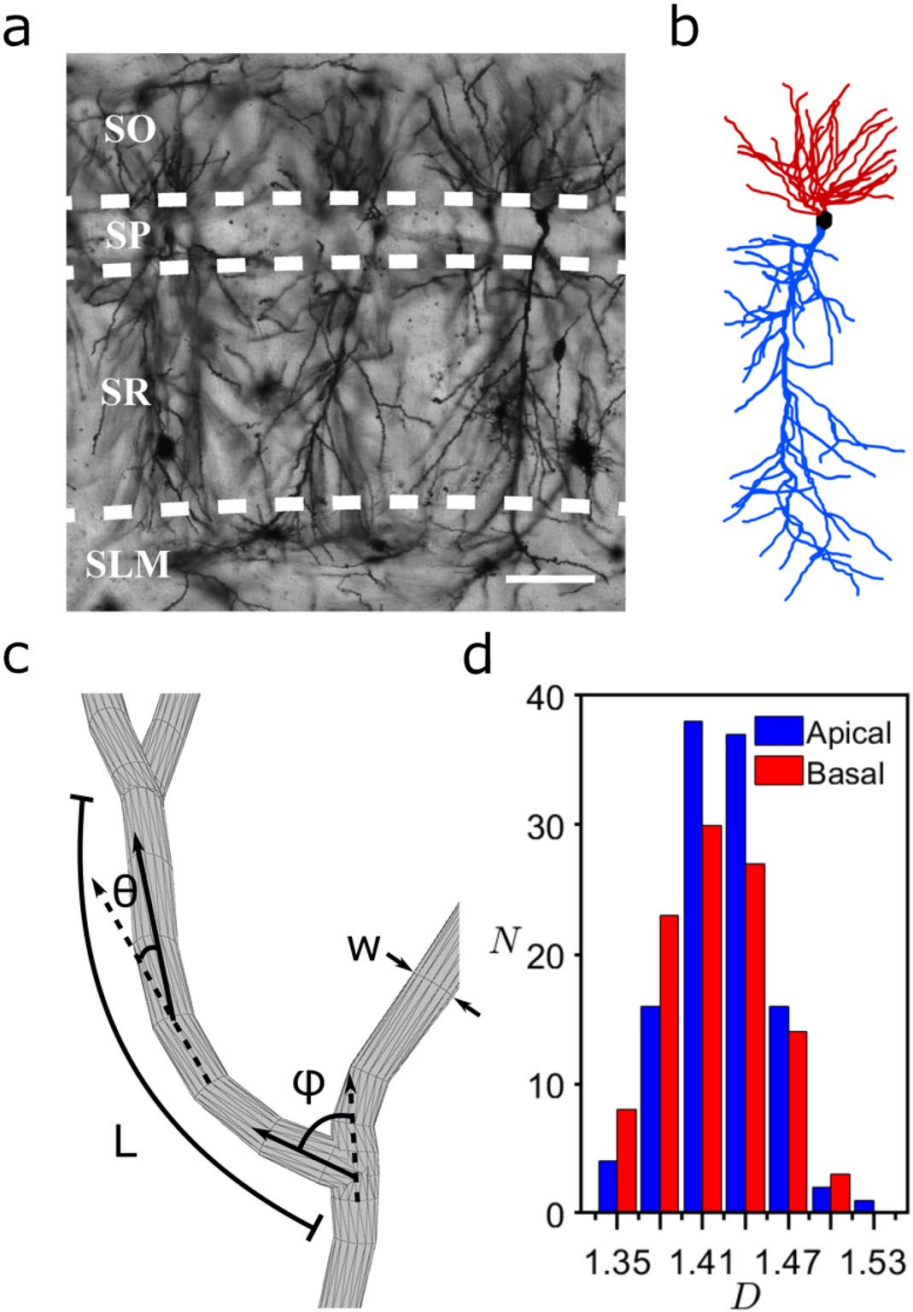
(**a**) An example confocal micrograph (x–y layer) showing three neighboring dendritic arbors, each spanning the oriens (SO), pyramidale (SP), radiatum (SR), and lacunosum-moleculare (SLM) strata of the CA1 region. The dashed lines represent the strata boundaries and the bar corresponds to 100 μm. (**b**) A three-dimensional model of a dendritic arbor (reconstructed from a stack of micrographs in the z direction using Neurolucida and displayed using MATLAB) featuring the apical (blue) and basal (red) arbors and the soma (black). The neuron’s axon arbor is not shown. (**c**) Schematic showing the neuron parameters *L*, *W*, *ϕ*, and *θ*. (**d**) Histogram of *N*, the number of neurons with a given *D* value, measured for the neurons’ apical and basal arbors.

We use confocal microscopy to obtain images of CA1 pyramidal neurons in the coronal plane of the dorsal rat hippocampus (Figs. 1a, 6). Their somata are located in the stratum pyramidale (SP) of the CA1 region. Axonal and dendritic arbors extend from each soma, with the dendritic arbor featuring component apical and basal arbors. The complex branching patterns of these dendritic arbors extend into the neighboring stratum radiatum (SR) and stratum oriens (SO) of the CA1 region where they collect signals from the axons of other neurons^26^. These axons originate either from within the CA1 region and connect to the dendritic arbors from every direction (e.g. O-LM cells, basket cells, bistratified cells, and axo-axonic cells)^37^, or they originate from other regions such as the neighboring CA2 which extends axons parallel to the strata (e.g. Schaffer collaterals). We construct three-dimensional models of the dendritic arbors (Fig. 1b) from the confocal images of ~100 neurons using Neurolucida software^38^ (“Methods”). The branches in the model are composed of a set of cylindrical segments which have a median length and width *W* of 2.4 μm and 1.4 μm, respectively (Fig. 1c). The branch ‘weave’ angles *θ* are defined as the angles between connecting segments along the branch. We define the fork angle *ϕ* as the first of the branch weave angles (Fig. 1c and “Methods”) for any branch not emanating from the neuron’s soma. The distinct median values for *θ *(12°) and *ϕ* (37°) motivated our approach of treating *ϕ *as a separate parameter from *θ*. Associated with each *ϕ *and *θ* value, there is an additional angle measuring the segment’s direction of rotation around the dashed axes in Fig. 1c. The branch length *L* is defined as the sum of segment lengths between the forks. As an indicator of arbor size, the maximum branch length *L*_*max*_ varies between 109–352 μm across all neurons, with a median value for *L/L*_*max*_ of 0.24. Because each parameter (*θ*, *ϕ*, and *L*) features a distribution of sizes (Fig. 7, Supplementary Fig. 1), we will investigate their potential to generate fractal branch patterns and consequently their impact on neuron wiring connectivity.

## Results

### Fractal analysis

In principle, a neuron could extend into the SR and SO layers following a straight line with dimension *D* = 1 or spread out and completely fill the space with a dimension of *D* = 3. If they instead adopt fractal branches, then these will be quantified by an intermediate *D* value lying between 1 and 3^1^. This fractal dimension quantifies the relative contributions of coarse and fine scale branch structure to the arbor’s fractal shape (fractals with larger contributions of fine structure will have higher *D* values than fractals with lower contributions of fine structure). Whereas a variety of scaling analyses have been applied to neurons^14,17,19,22,27,39–43^, here we adopt the traditional ‘box-counting’ technique to directly quantify their *D* value (“Methods”). This technique determines the amount of space occupied by the neuron by inserting it into a cube comprised of a three-dimensional array of boxes and counting the number of boxes, *N*_*box*_, occupied by the dendrites (Fig. 8). This count is repeated for a range of box sizes, *L*_*box*_. Fractal scaling follows the power law *N*_*box*_ ~ *L*_*box*_^*−D*^. Note that the box-counting analysis is applied to individual neurons and not to the network of multiple neurons: fractal networks often ‘space-fill’^44^ and it is the *D* values of their fractal elements that determine how they interact^11^. The histogram of Fig. 1d plots the number of neurons *N* with a given *D* value for both apical and basal arbors. The medians of their distributions are *D* = 1.41 (basal) and 1.42 (apical), indicating that their branches have similar scaling characteristics despite the apical arbors having longer branches that typically feature more forks. Given that *D* can assume values up to 3, it is intriguing that the dendrites’ *D* values are relatively low. Additionally, the scaling range over which the neurons can be described by this *D* value is limited to approximately one order of magnitude of *L*_*box*_ (“Methods”). This is inevitable because the coarse and fine scale limits are set by the widths of the arbor and its branches, respectively (“Methods”). We will show that this scaling behavior is so effective that its limited range is sufficient for the low *D* values to optimize the connectivity process. Accordingly, *D* serves as an ‘effective’ fractal dimension for quantifying neuron functionality despite lacking the range associated with mathematical fractal exponents.

**Figure 2:**
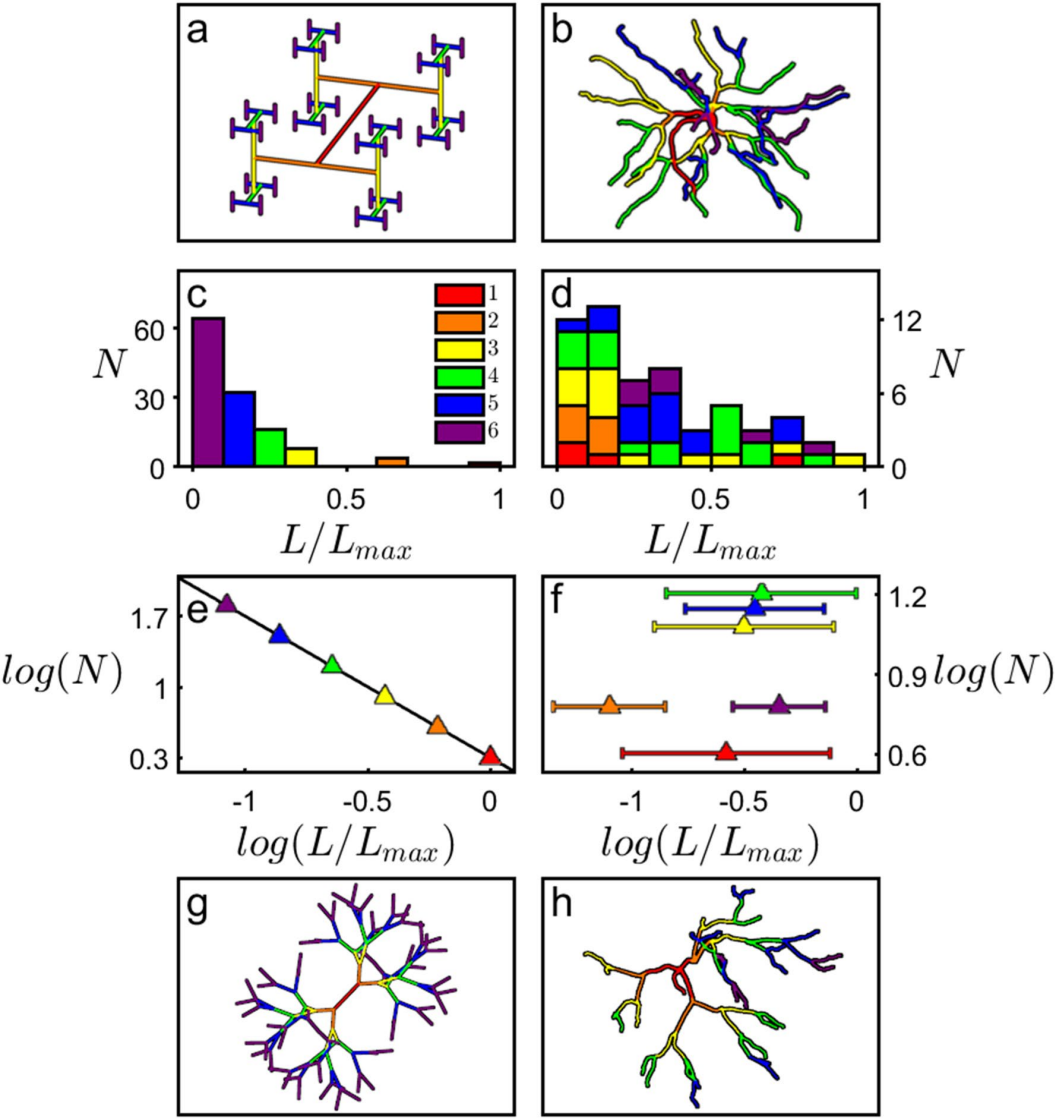
A *D* = 1.4 H-Tree fractal (generated using Mathematica and displayed using MATLAB) with *W* = 1 μm (**a**) and an example neuron’s basal arbor (reconstructed using Neurolucida and displayed using MATLAB) with median *W* = 1.4 μm (**b**). The branch level *i* is colored as follows: red (1st branch), orange (2nd), yellow (3rd), green (4th), blue (5th), and purple (6th). Histograms for an H-Tree (**c**) and neuron (**d**) plotting the number of branches *N* with a given value of *L/L*_*max*_. Panels (**e**) and (**f**) show the analysis of (**c**) and (**d**) plotted in log–log space. Panels (**g**) and (**h**) take the H-Tree and neuron shown in (**a**) and (**b**) and adjust all their branch lengths to be equal. Additionally, the H-Tree’s forking angle *ϕ* has been adjusted to 37° (the median value of the basal arbors).

To clarify this favorable functionality, we first need to determine which parameters (*θ*, *ϕ*, and/or *L*) contribute to the neuron’s fractal-like character. In mathematics, fractals can be generated by using forking angles (e.g. Self-contacting Trees, so named because their branch tips intersect), weave angles (e.g. Peano curves), or branch lengths (e.g. H-Trees)^1^. Because many mathematical fractals are generated by scaling *L*, we start by comparing the neurons’ *L* behavior to that of H-Trees (“Methods”, Supplementary Fig. 2). Figure 2 shows the scaling relationship of *N* (the number of branches with a given *L/L*_*max*_) measured for a *D* = 1.4 H-Tree (Fig. 2a,c,e) and a typical basal arbor (Fig. 2b,d,f). We assign the branch levels such that *i* = 1 corresponds to branches emerging from the soma, *i* = 2 to the branches emerging from the first forks, etc., with neurons featuring a median of 7 levels on the basal side and 24 levels on the apical side (other common level assignments such as the Strahler scheme^45^ generate similar findings to those below). The H-Tree exhibits the well-defined reduction in *L/L*_*max*_ as *i* increases (Fig. 2c) and follows the expected power law decrease in *N* as *L/L*_*max*_ increases (Fig. 2e): the magnitude of the data line’s gradient in Fig. 2e equals the H-Tree’s *D* value of 1.4. This behavior is absent for the neuron: in Fig. 2d, *L/L*_*max*_ does not exhibit a systemic reduction in *i* and consequently the Fig. 2f data does not follow a well-defined slope. Visual inspection of the equivalent Fig. 2d data for all of the neurons reveals no clear systematic dependence of their *L* distributions on *D* (6 representative neurons are shown in Supplementary Fig. 3). The neurons’ fractal-like character is even preserved when the *L/L*_*max*_ distribution is suppressed by setting all branch lengths equal (for the neuron shown in Fig. 2h, this common length is chosen such that the combined length of all branches matches that of the undistorted neuron of Fig. 2b). The median *D* value of the basal arbors drops from 1.41 to 1.30 during this suppression. This occurs because the lower branch levels of the undistorted neuron are consistently shorter than the higher levels^45^ (Supplementary Fig. 1). This characteristic is removed when the branch lengths are equated, so pushing the branches further apart and generating the drop in the ratio of fine to coarse structure seen in comparisons of Fig. 2b,h. Significantly, when we similarly suppress their branch length distribution, H-Trees with a sufficient number of levels exhibit the expected non-fractal behavior (*D* = 3) for *ϕ* = 90°, but display the limited-range fractal behavior if we instead assemble the H-Tree using the neurons’ median *ϕ *value (Fig. 2g). This highlights the important role of branch angles coupled with their length distributions for determining the fractal-like appearance.

**Figure 3:**
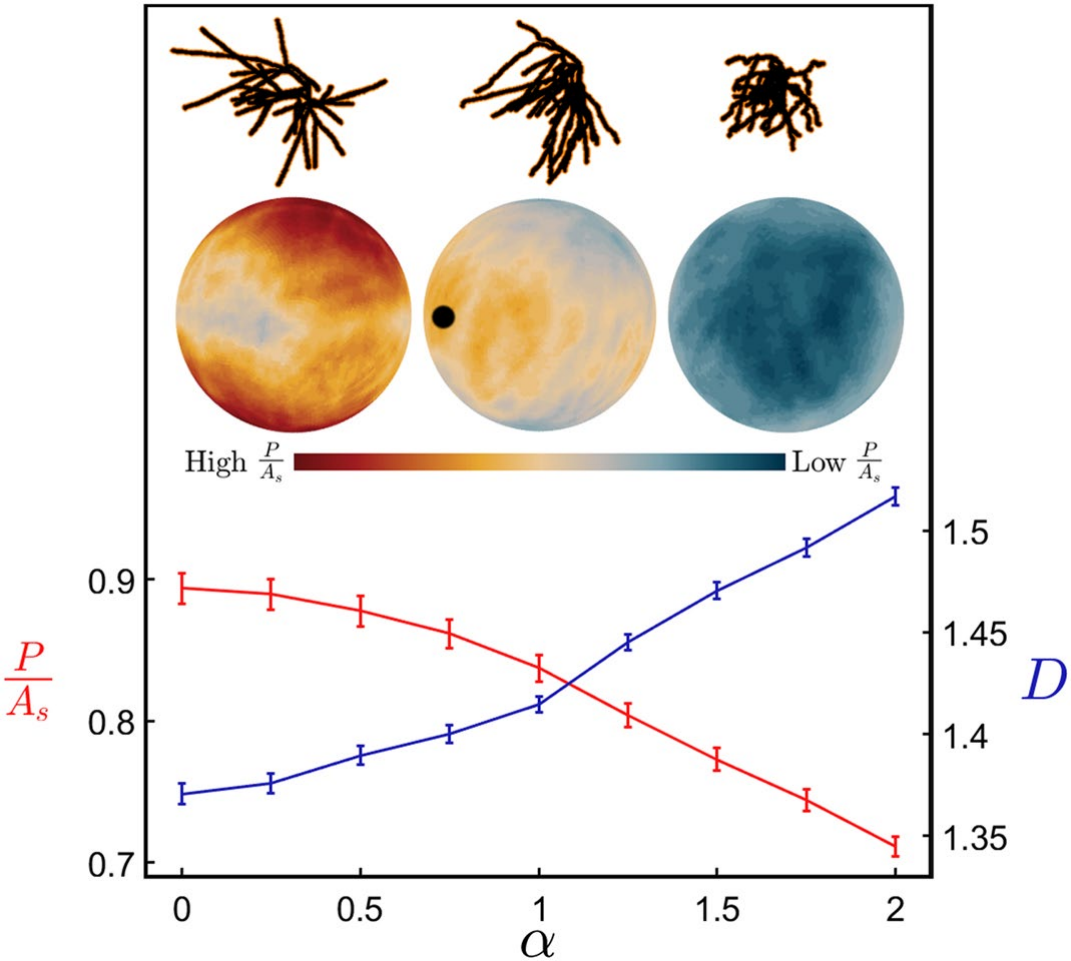
*P/A*_*s*_ (the arbor’s profile, *P*, averaged over all orientations and normalized to the arbor’s surface area, *A*_*s*_) (red) and fractal dimension *D* (blue) plotted against the weave angle manipulation factor *α* for the range of *α* resulting in physically reasonable model conditions. The data shown here for both the red and blue lines are averaged over all basal arbors and their variations are represented by the shown standard errors from the mean. The upper insets show an example neuron’s basal arbor for *α* = 0.25 (left), 1 (middle) and 1.75 (right). The neuron, reconstructed using Neurolucida, was altered and displayed using MATLAB. The lower insets show the equivalent profile spheres, where the black dot represents the orientation with maximal *P/A*_*s*_ for the middle neuron and the bar indicates the colors ranging from high to low *P/A*_*s*_ values.

This finding opens up an appealing strategy for exploring how the neuron’s *D* value influences its functionality. In Fig. 3, we mathematically manipulate the weave angles by multiplying every *θ* value by a common factor *α* (Fig. 7). This changes the neuron’s *D* value as follows. Values of *α* higher than 1 increase the weave angles above their natural values and cause the neuron branches to curl up. We set the highest value to be *α* = 2 to ensure that branches rarely intersect and penetrate, so ensuring a physically reasonable condition. As shown by the blue line in Fig. 3, this curling process causes the *D* value to rise because the amount of fine structure in the neuron’s shape increases. Similarly, reducing *α* causes the branches to gradually straighten out and this reduces the amount of fine structure and *D* drops. Figure 3 includes a visual demonstration of this curling process. Interestingly, a key feature of curling—that total branch length does not rise with *D*—is also displayed by the undistorted neurons (and deliberately incorporated into our H-Trees), further emphasizing the appropriateness of this technique. Applying this technique to *ϕ*, and also to *θ* and *ϕ *simultaneously (Supplementary Fig. 4), we find that either increasing or decreasing *α* results in a rise in *D.* This is because the branches self-avoid at *α* = 1 and so move closer together when *α* is either increased or decreased. This generates an increase in the ratio of fine to coarse structure. Plots of *D* directly against the means of *θ* and *ϕ* are shown in Supplementary Fig. 5.

### Connectivity analysis

We now investigate the impact of changing *D* on the neuron’s potential to connect to other neurons. Previous studies established that the arbor’s physical structure is sufficient for describing the connection process, with chemical steering playing a relatively minor role^46,47^. In particular, the arbor’s dendritic density^33,48–50^ and resulting physical profile^27^ are powerful indicators of its potential to connect to other neurons. When viewed from a particular orientation, we define the arbor’s profile *P* as the total projected area of its branches. Large profiles will therefore result in the increased exposure of synapses, which are responsible for receiving signals from other neurons. When calculating the profile from the dendrite images, we incorporate an extra layer (orange in Fig. 3 upper inset, Supplementary Fig. 6) surrounding the branches (black) to account for outgrowth of dendritic spines—small protrusions which contain the majority of the dendrite synapses (“Methods”). For each arbor shown in Fig. 3, *P* is therefore the sum of the projected black and orange areas. We then normalize this projected surface area of the dendrites using their total surface area, *A*_*s*_, to accommodate for the range in neuron sizes and associated surface areas. (Because the orange areas are included in *P* but not in *A*_*s*_, note that *P/A*_*s*_ > 1 is possible). The current study adopts the general approach of averaging *P/A*_*s*_ across all orientations of the dendritic arbor to allow for the fact that axons originating from within the CA1 region connect to the dendritic arbors from every direction^37^. The profile variation with orientation can be visualized by projecting the *P/A*_*s*_ values obtained for each direction onto a spherical surface. For the profile spheres included in Fig. 3, the neurons are viewed from a common direction which corresponds to the middle point on the sphere’s surface. For the natural neuron, the orientation for which *P/A*_*s*_ peaks is marked by the black dot. Typically, this peak occurs in the direction that the Schaffer collateral axons enter from the CA2 region^26^ and so maximizes the connectivity of our natural neurons to those incoming axons.

**Figure 4:**
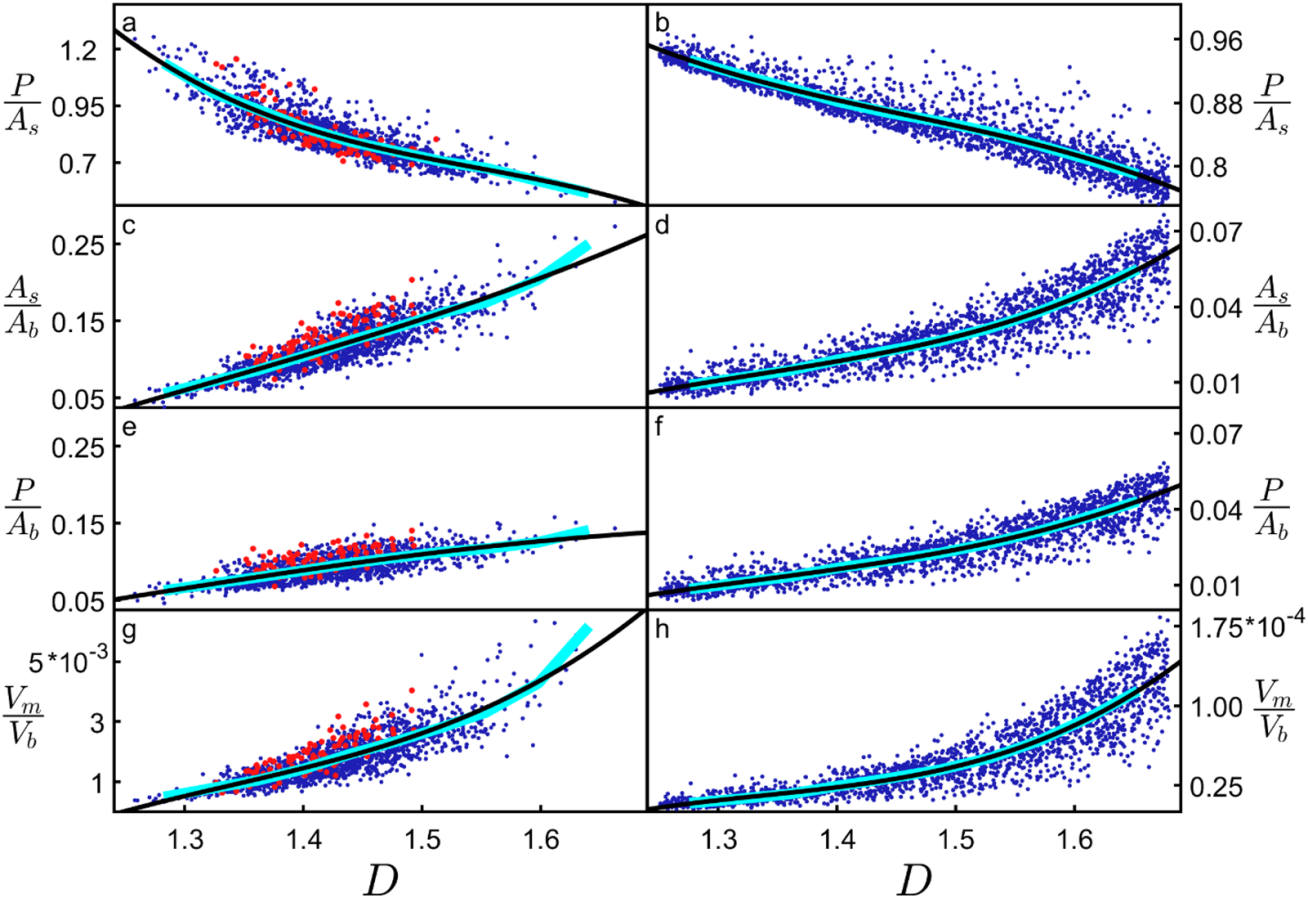
Dependences of various parameters (see text for parameter definitions) on *D* for neurons (left column) and H-Trees (right column). Red data are for unmanipulated basal arbors while the blue data are for basal arbors where either their *θ* or *ϕ *values are manipulated. H-Trees with straight and with weaving branches are included. The cyan lines correspond with binned averages of the plots while the black curves correspond to 3rd degree polynomial fits to the data.

The inverse relationship between *P/A*_*s*_ and *D* observed when adjusting the weave angle in Fig. 3 also occurs when adjusting the fork angle (Supplementary Fig. 4). Its physical origin can be traced to the increased fine structure of high *D* neurons causing branches to block each other and so reduce the overall profile. Including this blocking effect is important for capturing the neuron’s connectivity because multiple connections of an axon to the same dendritic arbor are known to generate redundancies^27^. Therefore, if a straight axon connected to an exposed branch, subsequent connections to blocked branches wouldn’t increase the connectivity and should be excluded. Figure 4a summarizes this blocking effect by plotting *P/A*_*s*_ directly against *D* for arbors that have had their *θ* and *ϕ *values manipulated independently. Figure 4b demonstrates that this blocking reduction in *P/A*_*s*_ is also seen for H-Trees (which have had their weaves similarly adjusted—see “Methods” and Supplementary Fig. 2), highlighting that the blocking dependence on *D* is general to fractals. Figure 4c,d explore another well-known fractal effect that high *D* fractals increase the ratio of the object’s surface area *A*_*s*_ to its bounding area *A*_*b*_^1,2^ (i.e. the surface area of the volume containing the arbor, as quantified by its convex hull—see “Methods”). Figure 4e,f combines the ‘increased surface area effect’ seen in Fig. 4c,d with the ‘blocking area effect’ seen in Fig. 4a,b by plotting *P/A*_*b*_ (i.e. the multiplication of *P/A*_*s*_ and *A*_*s*_*/A*_*b*_) against *D*. In effect, *P/A*_*b*_ quantifies the large surface area of the arbor while accounting for the fact that some of this area will be blocked and therefore excluded from the profile *P*. Normalizing *P* using *A*_*b*_ serves the additional purpose of measuring the arbor’s potential connectivity in a way that is independent of its size. Accordingly, *P/A*_*b*_ serves as a connectivity density and is an effective measure of the neurons’ capacity to form a network.

The clear rise in *P/A*_*b*_ revealed by Fig. 4e,f highlights the functional advantage offered by high *D* branches—incoming axons will experience the dendritic arbor’s large connectivity density. Note that the plotted connectivity density is for individual neurons. Because of the inter-penetrating character of dendritic arbors from neighboring neurons, the collective connectivity density will be even larger due to their combined profiles. If this functionality was the sole driver of neuron morphology, then all neurons would therefore exploit high *D* values approaching 3. Yet, both the apical and basal dendrites cluster around relatively low values of *D* ~ 1.41 suggesting that there are additional, negative consequences of increasing *D*. In Fig. 4g,h, we plot the ratio of the volume occupied by the branches *V*_*m*_ to the neuron’s bounding volume *V*_*b*_ (i.e. the arbor’s convex hull volume). For high *D* dendrites, the tighter weave angles along with forking angles that bring branches closer together result in more densely packed structures. This produces the observed rise of *V*_*m*_*/V*_*b*_. Assuming constant tissue density, *V*_*m*_ is proportional to the neuronal mass. The rise in *V*_*m*_*/V*_*b*_ therefore quantifies the increase in mass density and associated ‘building’ costs of high *D* neurons. Aside from this, there is also an ‘operational’ cost. It is well-known from allometric scaling relationships that metabolic costs generally increase with mass^8,10^. Specifically, previous research proposed that the amount of ATP expended by neurons increases with *A*_*s*_^27,30^. Revisiting Fig. 4a,c, *A*_*s*_*/A*_*b*_ therefore charts how the normalized energy cost increases with *D*, and *P/A*_*s*_ measures the neuron connectivity relative to this cost. In Figs. 3 and 4, we didn’t apply the *α* multiplication technique to the neurons’ distribution of *L* values because this would simply change the size of the arbors and have no impact on their fractal characteristics.

## Discussion

**Figure 5:**
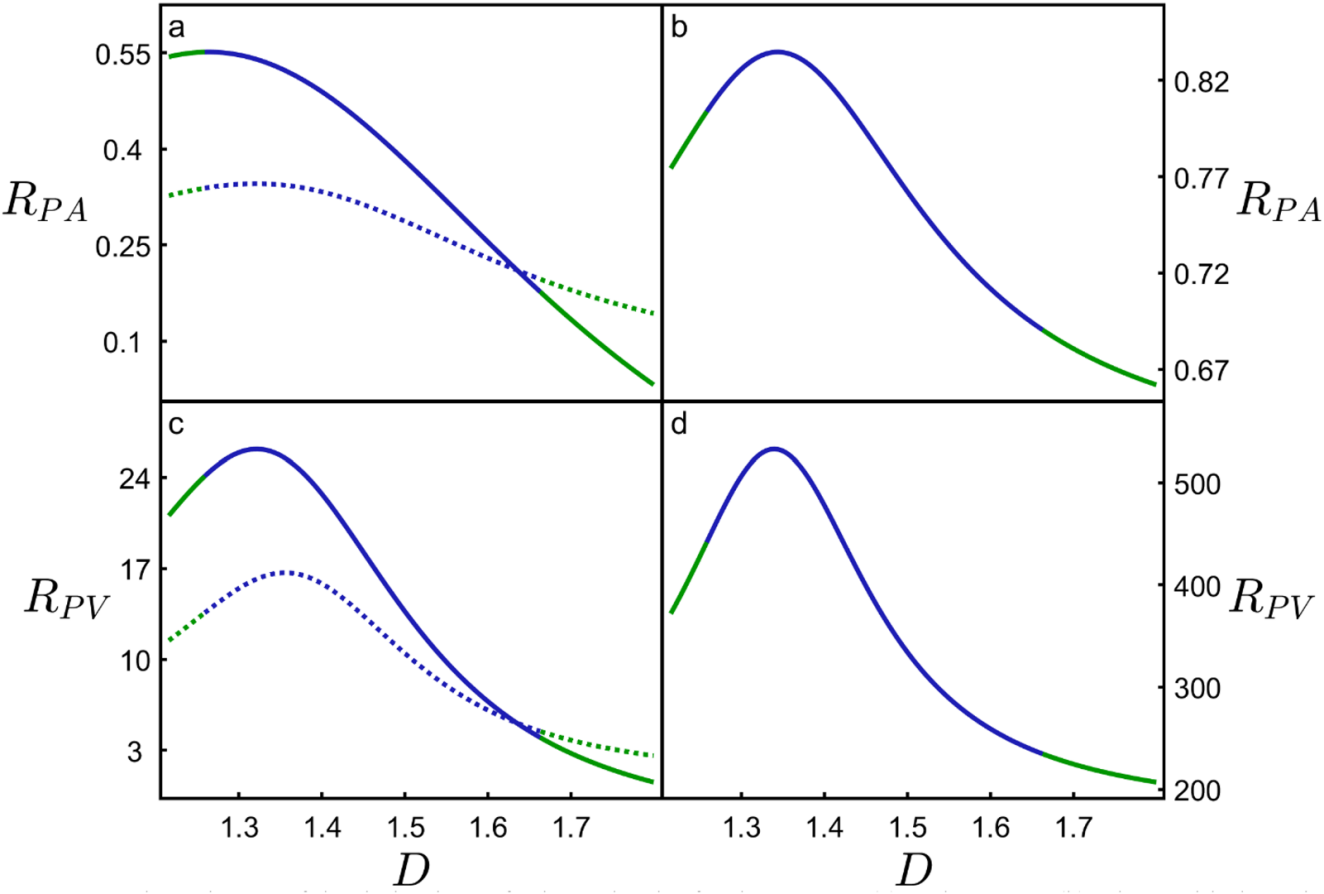
The ratio *R*_*PA*_ of the derivatives of *P/A*_*b*_ and *A*_*s*_*/A*_*b*_ for the neurons (**a**) and H-Trees (**b**), along with the ratio *R*_*PV*_ of the derivatives of *P/A*_*b*_ and *V*_*m*_*/V*_*b*_ for the neurons (**c**) and H-Trees (**d**). The blue sections correspond to the data range shown in Fig. 4, while the green sections correspond to extending the fits to beyond that data range. The dashed curves in (**a**) and (**c**) indicate the effect on *R*_*PA*_ and *R*_*PV*_ of removing the dendritic spines from the model. The peaks of the solid curves in (**a**), (**b**), (**c**), and (**d**) occur at *D* = 1.26, 1.34, 1.32, and 1.34, respectively. The peaks of the dashed curves in (**a**) and (**b**) occur at *D* = 1.32 and 1.36, respectively.

Taken together, the panels of Fig. 4 summarize the competing consequences of increasing *D* for both the neurons and H-Trees: the benefits of enhanced connectivity density increase (Fig. 4e,f), but so does the cost of building (Fig. 4g,h) and operating (Fig. 4c,d) the branches. The distinct forms of these 3 factors are highlighted using 3rd degree polynomial fits (black) which closely follow the binned average values of the data (cyan). This observation of neuron behavior across large *D* ranges provides a clear picture of their tolerances for the above factors and highlights both the shared behavior and subtle differences to standard mathematical fractals such as H-Trees. In particular, the high operating cost, the sharp increase in building cost, and the flatter gradient of the connectivity curve at high *D* could explain why the natural neurons (red) don’t exceed *D* = 1.51. Nor do they occur below *D* = 1.33 because of the low connectivity. To explore how the fractals balance these factors, in Fig. 5 we consider the ratios of the rates of change of connectivity density with operating cost

$${R}_{PA}=\frac{\frac{d}{dD}\left(\frac{P}{{A}_{b}}\right)}{\frac{d}{dD}\left(\frac{{A}_{s}}{{A}_{b}}\right)}$$

and with building cost

$${R}_{PV}=\frac{\frac{d}{dD}\left(\frac{P}{{A}_{b}}\right)}{\frac{d}{dD}\left(\frac{{V}_{m}}{{V}_{b}}\right)}$$

as simple optimization indicators. An in-depth comparison of the four curves in Fig. 5 is limited by the fits in Fig. 4 (in particular, the data scatter and the fact that we can’t increase the *D* range without resorting to *α* values that result in physically unreasonable model conditions, see earlier). Nevertheless, we note the following shared characteristics: (1) the occurrence of a peak at low *D*, (2) *R*_*PA*_ < 1 (i.e. the connectivity increases more slowly than the energy costs), and (3) *R*_*PV*_ > 1 (the connectivity increases more rapidly than the mass costs). Consequently, although high *D* offers superior connectivity for the neurons, the positive consequences of increasing *D* beyond the peak rapidly diminish in terms of the mass costs of establishing connectivity. Simultaneously, the negative consequences of increasing *D* rapidly rise in terms of the energy costs of establishing connectivity. Figure 5a,c includes a comparison of our model including and excluding the spine layer to highlight the sensitivity of the curves to connectivity. As expected, removal of the spines triggers an overall drop in *R*_*PA*_ and *R*_*PV*_, and their peaks shift to higher *D* values in an attempt to regain connectivity. Intriguingly, the peaks in Fig. 5a,c suggest an optimized *D* value lower than the natural neurons’ prevalent *D* value (for example, *R*_*PV*_ peaks at *D* = 1.32 compared to *D* = 1.41 in Fig. 1d). Given that the spine layer width is the only input parameter to the model and its complete exclusion is insufficient to raise the optimization values up to *D* = 1.41, Fig. 5 suggests that the optimization process places a greater emphasis on connectivity than our simple ratios would suggest. A previous study^27^, which limited its focus to the optimization condition, also under-estimated the neuron scaling exponent slightly (1.38), although we note that the 2 exponents are not identical—their exponent characterized small sections of the arbor compared to the overall arbor *D* presented here. Given that other fractal studies of neurons employed additional scale-invariance measures such as multifractal dimensions and lacunarity^6^, our future studies will expand the approaches of Figs. 4 and 5 to determine if including these measures allows for a more accurate prediction of the peak values.

Based on our analysis spanning large *D* ranges beyond their naturally occurring conditions, we hypothesize that different neuron types have different *D* values depending on the relative importance of connectivity and cost. Neurons with a greater need for connectivity will optimize around higher *D*. For example, human Purkinje cells are characterized by *D* ~ 1.8^18^. We also hypothesize that pathological states of neurons, for example those with Alzheimer’s disease, might affect the fractal optimization and explain previous observations of changes in the neurons’ scaling behavior^51^. Whereas the above discussions focus on connections to neighboring neurons, their fractal branching will also optimize connections to neighboring glial cells to maximize transfer of nutrients and energy. Intriguingly, CA1 hippocampal glial cells have been shown to have similar *D* values to the neurons in our study (*D* = 1.42)^52^.

Fractal analyses of a wide variety of neurons indicate that their *D* values don’t generally exceed *D* = 2^17,23^, presumably because of the excessive costs of higher *D* fractals. For comparison, we note that a sphere (*D* = 3) achieves much higher connectivity (*P/A*_*b*_ = 0.25 compared to the *D* = 1.4 neuron’s 0.1). However, the sphere suffers from large mass density (*V*_*m*_*/V*_*b*_ = 1 compared to 10^–3^) and higher operational costs (*A*_*s*_*/A*_*b*_ = 1 compared to 0.1), suggesting that neurons adopt fractal rather than Euclidean geometry in part because the mass and operational costs of the latter are too high. We note that neurons’ restriction to lower *D* values doesn’t apply to fractal electrodes designed to stimulate neurons^53–55^. These artificial neurons require large profiles to physically connect to their natural counterparts. However, unlike natural neurons, the large *A*_*s*_ associated with high *D* electrodes reduces the operation costs because their higher electrical capacitances lead to larger stimulating electric fields^53,54^. Thus, fractal electrodes approaching *D* = 3 might be expected to efficiently connect to and stimulate neurons. That said, there might be advantages of matching the electrode’s *D* value to that of the neuron: this will allow the neuron to maintain its natural weave and forking behavior as it attaches to and grows along the electrode branches, so maintaining the neuron’s proximity to the stimulating field.

Previous studies of connectivity and dendritic cost focused on component parameters of the neuron geometry such as tortuosity (which quantifies branch weave using, for example, a simple ratio of branch length to the direct distance between the branch start and end points), branch length, and scaling analysis of small parts of the arbor^27,33^. We have shown that our ‘effective’ *D* incorporates these parameters in a holistic approach that directly reflects the fractal-like geometry across multiple branches of the neuron’s arbor. For example, our discovery that the neurons’ weave (generated by variations in *θ* and *ϕ*) is an important factor in determining *D* provides a link between *D* and tortuosity. However, whereas tortuosity quantifies the weave of an individual branch measured at a specific size scale, *D* captures a more comprehensive picture by accounting for the weave's tortuosity across multiples scales. Future studies will examine the precise relationship between *D* and the scaling properties of tortuosity for both individual branches and the whole arbor.

Because *D* is sensitive to 3 major branch parameters (*θ, ϕ*, and *L*), it is a highly appropriate parameter for charting the connectivity versus cost optimization discussed in this *Article*. Whereas we have focused on this *D*-dependent optimization, the future studies will further examine the intricacies of how the branch parameters determine* D* and *T*. Here, we have seen that the distorted neurons show systematic dependencies of their mean *D* values on *θ *and *ϕ* (Supplementary Fig. 5) and a drop in *D* when their branch lengths are equalized (Fig. 2h). We emphasize, however, that the interplay of these 3 dependences generates variations in the individual neurons’ *D* values and these are responsible for the spread in *D* observed for the natural neurons (Fig. 1d). Their *D* values do not exhibit any clear dependence on *θ *and *ϕ* when plotted independently (Supplementary Fig. 5) and, as noted earlier, the spread in *D* does not display any systematic dependence on their *L* distributions. It is not clear if these *D* variations between natural neurons are simply obscuring the systematic behavior followed by the distorted neurons or whether, more intriguingly, the natural neurons are using this interplay of the three dependences to anchor their *D* values around the narrow range that results in the optimization condition. The future investigations aim to illuminate this interplay.

Finally, our focus on *D* to investigate neurons facilitates direct comparisons with the favorable functionalities generated by diverse structures. Here, we compared our neurons to distorted versions, to H-Trees, to fractal electrodes, and to Euclidean shapes, but this approach could readily be extended to many natural fractals. The fact that the H-Trees and neurons exploit a similar *D*-dependent optimization process (Figs. 4 and 5) raises the question of why the two structures use different branch length distributions (Fig. 2) to generate their scaling behavior. The answer lies in the neuron’s need to minimize signal transport times within the arbor^56^. This is achieved with short branches close to the soma (Supplementary Fig. 1) while the H-Tree suffers from longer branches. Remarkably, Figs. 4 and 5 show that the *D*-dependent behavior impacts neuron functionality even though it occurs over only a limited range of branch sizes. Many physical fractals are also limited^57^, demonstrating the effectiveness of fractal-like behavior for optimizing essential processes ranging from oxygen transfer by our lungs, to light collection by trees, to neuron connections throughout the body. For neurons, we have shown how they use this limited fractality to balance wiring connectivity with material, energy, volume, and time (signal) costs.

## Methods

### Rodents

The study was conducted in accordance with ARRIVE guidelines. Rat pups were bred and housed with their mother in cages with wood chips and ad libitum food and water in an environmentally controlled room. All procedures pertaining to the use of live rats were conducted in compliance with all relevant ethical regulations for animal testing and research and were approved by the University of Canterbury Animal Ethics Committee, 2008-05R.

### Image acquisition and model reconstruction

**Figure 6:**
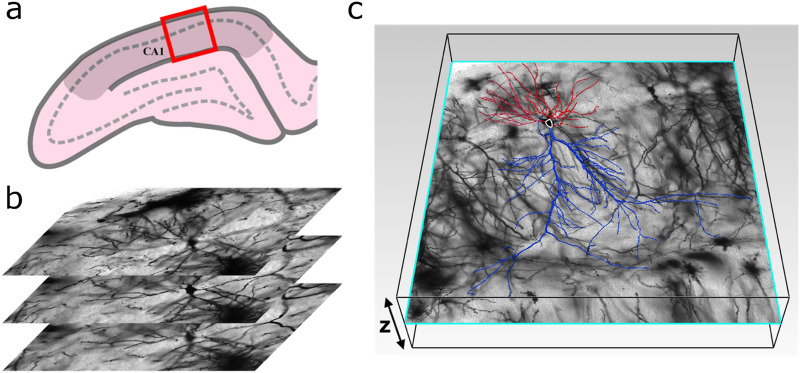
(**a**) Schematic diagram of a coronal slice through the hippocampus at Bregma −4.52 mm showing the collection region (red box) within hippocampal CA1 (darkened area); the somata layer is denoted by the dashed line. (**b**) Confocal micrographs of Golgi-Cox stained cells. Three 774 by 774 µm cross-sections separated by 2 µm in the z-direction are shown. (**c**) A model showing a neuron’s soma (outlined in white) as well as its basal (red) and apical (blue) dendritic arbors superimposed on the original micrograph. The image in (**c**) was generated using Neurolucida.

Thirty-three adult PVGc male hooded rats (13–16 months old) were given an overdose of sodium pentobarbital. The brains were removed fresh without perfusion, rinsed with Milli-Q water, and a 4 mm block containing the hippocampus was cut in the coronal plane using a brain matrix (Ted Pella, Kitchener, Canada). These tissue blocks were processed with a metallic Golgi-Cox stain, which stains 1–5% of neurons so that their cell bodies and dendritic trees can be visualized. Thick 200 µm coronal brain sections spanning the bilateral dorsal hippocampus were taken using a microtome. A standard microscope was used to locate isolated neurons in the dorsal CA1 subfield (Fig. 6a). These large pyramidal neurons consist of a long apical dendritic tree protruding from the apex of the soma and a shorter basal dendritic tree protruding from the other end (Fig. 1b). Only some intact whole neurons were located, whereas many intact basal-only or apical-only dendritic trees were located. In total, 105 basal and 113 apical arbors were imaged. A Leica laser scanning confocal microscope was used to collect high-resolution image stacks for each of these neuronal processes (Fig. 6b). The image stacks were captured using a 20 × glycerol objective lens with a 0.7 numerical aperture, providing an x and y resolution of 0.4 µm. The step size (z distance between image stacks) was 2 µm. Dendritic arbors were manually traced through the image stacks using Neurolucida^38^ (MBF Bioscience, Williston, VT, USA) to create three-dimensional models (Figs. 1b, 6c). The models were then exported to the Wavefront .obj format and the cell soma removed. The analysis of these models was done by authors of this study that were blinded to rat ID numbers.

The three-dimensional models are composed of a set of connected hollow cylinders (segments) which form the branches of the arbors. Each cylinder is constructed using two sets of rings of 16 points (vertices) and 32 connecting triangles (faces). At branch endings, the final segment has 14 faces that form an end cap. Connecting branches start new segments at the same location as the last set of 16 vertices from the previous branch. For the apical arbors, one branch extends from the apex of the soma and all other branches connect either directly or indirectly to it. For the basal arbors, multiple initial branches extend from the soma, each with its own set of connecting branches.

In order to perform the box-counting and profile analyses, the Wavefront files were converted to voxel data using custom MATLAB code. The voxelization was performed at a resolution of 4 voxels/µm for box counting and 1 voxel/µm for the profile calculation. In both cases, the models were voxelized “exactly,” meaning that if any part of the polygonal model fell inside a voxel, the voxel was added to the list of x; y; z coordinates.

**Figure 7:**
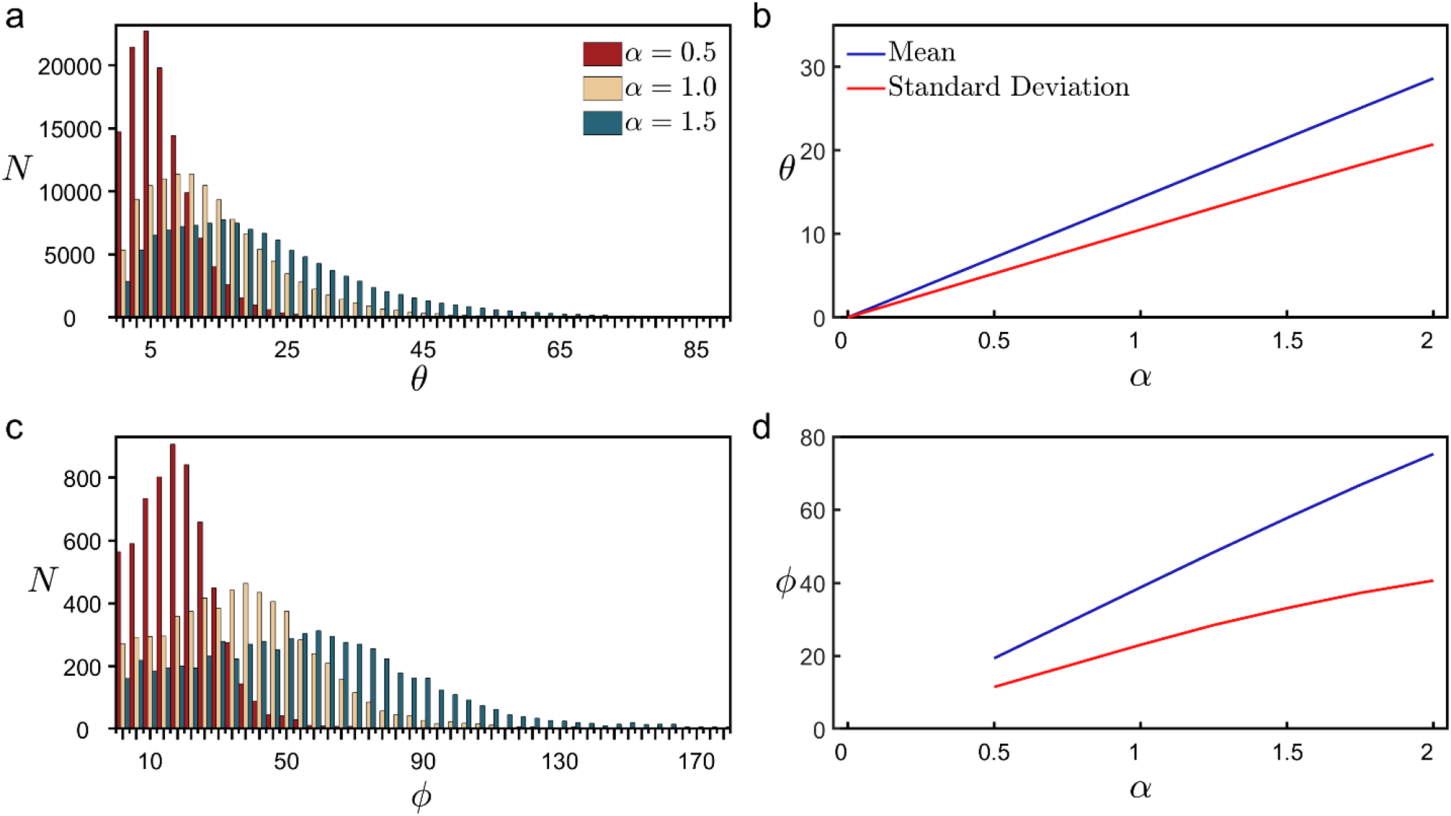
Histograms of the number *N* of occurrences of weave (**a**) and fork (**c**) angle values across all basal arbors used in the study. The impact of modifying these angles is shown for three *α* values. (**b**,**d**) Changes to the peak and breadth of the distribution of these angles as a function of *α* shown for the range of *α* that results in physically reasonable model conditions (see Fig. 3 and Supplementary Fig. 4).

We used rotation quaternions^58^ to adjust the weave and fork angles to the modified values multiplied by the pre-factor *α* When adjusting the weave angles, we started with the angles furthest from the soma and, working inwards, adjusted angles one at a time until all of the angles had acquired their new values. When an angle was adjusted, the entire connected section of the branch between that angle and the terminal endcaps was also rotated. This rotation occurred in the plane of the two vectors that define that angle. We created three sets of arbor models modified by the multiplier *α* for values between 0 and 2, incremented by 0.25. In one set *θ* was modified, in a second set *ϕ *was modified, and in a third set both were modified simultaneously. The effect that the multiplier *α* has on the distribution of weave and fork angles can be seen in Fig. 7. Because our investigation focused on weaving and forking deviations relative to the dashed lines shown in Fig. 1c, their measured angles do not distinguish between whether the branches fall to the left or right of these lines. Accordingly, for the rare examples when the *α* multiplication generated angles greater than 180° (corresponding to branches crossing over the dashed line) the angles were adjusted so that they remain within the range between 0° and 180°. For example, when *α* = 2 was applied to a natural forking angle of 100°, the resulting angle was measured as 160° rather than 200°. This measurement scheme caused the slight non-linearity seen at large *α* values for the red and blue curves of Fig. 7d. The percentages of weave angles crossing the dashed line were 0.009% and 0.07% when *α* = 1.5 and 2, respectively. The corresponding percentages for the forking angles were 0.7% and 2.7%. We note that the measurement scheme did not impact the conclusions of this *Article*. For completeness, we also note that if we did not distinguish between the forking and weave angles and instead treated them as one set of angles, the combined distribution of angles would follow a similar behavior to that shown in Fig. 7a,b because of the relatively small number of forking angles.

When calculating the surface area, *A*_*s*_, of the models, the precision was raised by increasing the number of faces in a neuron’s construction four-fold by converting each triangle into four sub-triangles using a midpoint method (Supplementary Fig. 7). We then summed the areas of all the triangles in the model, excluding the faces where all three vertices resided inside another segment. The bounding area, *A*_*b*_, and bounding volume, *V*_*b*_, were calculated using the convex hull method^59^ on the vertices of the Wavefront object. *A*_*b*_ is the sum of the areas of all triangles composing the convex hull that encloses the vertices, whereas *V*_*b*_ is the volume enclosed within those triangles.

### Box counting analysis

**Figure 8:**
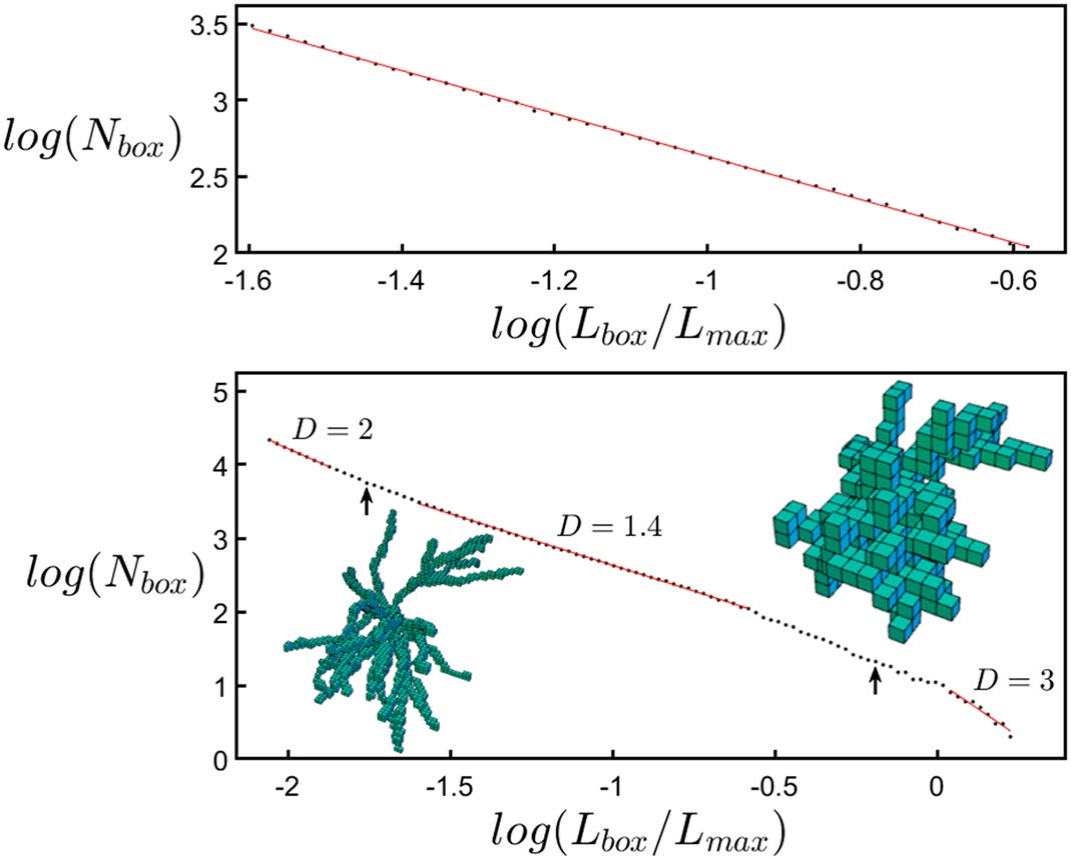
*log(N*_*box*_*)* plotted against *log(L*_*box*_*/L*_*max*_*)* for an example neuron’s dendritic arbor, where *N*_*box*_ is the number of occupied boxes and *L*_*box*_ is the box length. The top graph shows a zoom-in on the fractal-like scaling region of the bottom graph. The insets show occupied boxes at small and large box scales. See the text for explanations of the arrows.

The box-counting method used to analyze the fractal characteristics of the neurons is shown in Fig. 8. Using custom MATLAB code, the voxelized dentritic arbor was inserted into the three-dimensional array of boxes and the number of boxes *N*_*box*_ occupied by the neuron were counted for different sizes of boxes, *L*_*box*_, and this was normalized to *L*_*max*_, the largest branch size of the arbor. The largest box size was set to the size of the longest side length of the arbor’s bounding box and the smallest box size to the voxel pixelization (0.25 µm). The insets show example schematics of the filled boxes for large and small *L*_*box*_ values. We performed a modified ‘sliding’ box count^13^ in which the boxes slid in every coordinate direction simultaneously in 0.25 µm steps and the minimum count was selected.

Fractal scaling, *N*_*box*_ ~ *L*_*box*_^*-D*^, appears as a straight line in the log–log plot. The range of *L*_*box*_*/L*_*max*_ values for which fractal behavior might be observed lies between the vertical arrows. For large boxes to the right of the right arrow, there are too few boxes (less than five along each side of the bounding box) to reveal the fractal behavior. Consequently, all of the boxes become filled and so the analysis interprets the neuron as being three-dimensional and the gradient eventually shifts to a value of three. To the left of the left arrow (corresponding to 2 μm), the box sizes approach the diameters of the branches and so the analysis starts to pick up the two-dimensional character of the branches’ surface and the gradient shifts to a value of two.

Between the arrows, a straight line was fitted for all sets of points ranging over at least one order of magnitude. The fit that maximized *R*^2^ was chosen to measure the *D* value (the slope of the line). When looking at the residuals of this regression analysis, their behavior confirmed that the fit range was appropriate. We note that applying the angle multiplier to the neurons didn’t reduce the quality of the fit nor the scaling range of fractal behavior.

### Profile analysis

An arbor’s physical profile^27^ has been shown to be intrinsically related to its ability to connect with other neurons. We developed custom MATLAB code that measures the profile of the dendritic arbor using a list of cartesian points in space that denote the locations occupied by the dendrites. In our calculations, we used the voxelized list of points generated using the Wavefront file of the dendritic arbor. To allow the dendritic spines to contribute to the calculation of an arbor’s profile, we uniformly expanded the voxelized arbor by 2 µm in every direction. The orange region around the black dendrites seen in Supplementary Fig. 6 represents the space around the dendrites in which a spine could grow in order to form a synapse with an axon passing through the arbor. Including a solid orange region assumes a high spine density. Lower densities could be accommodated by reducing the profile by a density pre-factor. Given the comparisons in the current study are made across the same neuron type with the same spine density, the value of the pre-factor will not impact the results.

This expanded list of points was then orthographically projected onto the x–y plane. After projection, the points were rounded and any duplicate points occupying the same location were removed. Because the location of the points has been rounded, each point represents a 1 µm^2^ area and the total area occupied by this projection can be measured by counting the number of remaining points constituting the projection. The area of this projection divided by the bounding area of the neuron is then proportional to the probability of connection with an axon travelling parallel to the z-direction and passing through the dendritic arbor. However, because the axons that pass through the arbors of our CA1 neurons can arrive from any direction^26,37^, we calculated the average profile of each neuron’s arbor as though it were viewed from any point on the surface of a sphere containing the neuron’s arbor with its origin at the neuron’s center of mass. To accomplish this, we defined a set of polar and azimuthal angles that corresponded to uniformly distributed viewpoints on the sphere. By rotating the expanded list of points by these angles and then projecting the result onto the x–y plane, we obtained what the arbor would look like if seen from the given viewpoint. We calculated the average profile by averaging the area of the projections corresponding with each of our uniformly distributed viewpoints. Because it is impossible to distribute a general number of equidistant points on the surface of a sphere^60^, we defined our set of points using the Fibonacci lattice, a commonly used and computationally efficient method for distributing the points^61^.

The colored spheres (comprised of 10,001 points) in Fig. 3 and Supplementary Fig. 6 give a visual representation of the variation in profile with respect to the viewpoint. The average profile data used in Fig. 4 was calculated using only 201 viewpoints, which is sufficient for convergence—the average *P* for 201 viewpoints deviates by less than 1% from the value achieved when approaching infinite viewpoints.

### H-Tree generation

We compare the neurons to H-Trees to identify the similarities and differences to a traditional mathematical fractal pattern in which *D* is set by scaling the branch length *L* (we caution that any similarities do not imply a shared growth mechanism^44^). Supplementary Fig. 2 shows examples of the H-Tree models used to generate the data of Fig. 4b,d,f. Whereas these H-Trees extend into three-dimensional space (middle and bottom row), we also include two-dimensional H-Trees (top row) for visual comparison. Using Mathematica software, the *D* values of these straight branched models were generated using the branch scaling relationship

$${L}_{i}=\frac{{L}_{1}}{{2}^{\frac{i-1}{D}}},$$

where ${L}_{i}$ is the branch length of the *i*th level. The H-Trees used to generate the data seen in Fig. 4 had 12 levels of branches and the length of the first branch, ${L}_{1}$, of any given H-Tree was chosen such that the total length of all the branches was constant across all *D* values. For comparison of the H-Trees with the basal arbors in Fig. 4, the number of branch levels in the H-Tree was chosen to be close to the largest number of levels observed for the basal arbors (11) and the width of the H-Trees branches was chosen to be 1 μm which is similar to the 1.4 μm median width of the branches for the basal arbors. The *D* values of H-Trees in the bottom row of Supplementary Fig. 2 are determined by a combination of the length scaling between branch levels and the weave of the branches. The distribution of weave angles was generated using a fractional gaussian noise process, which is known to be self-similar, and the resulting *D* values were measured using the box-counting algorithm. The width of the weave angle distribution was specified before generating the H-Tree, allowing for fine control over the tortuosity of its branches. By using four different weave angle distribution widths and creating H-Trees with a multitude of *D* values, we demonstrated the robustness of the relationship between the *D* value and our various functional parameters shown in Fig. 4.

## Supplementary Information

Supplementary Figures.

## Data Availability

All data necessary to reproduce the results are available at https://github.com/jsmith767/NeuronFractalGeometry.

## Code availability

All scripts necessary to reproduce the results are available at https://github.com/jsmith767/NeuronFractalGeometry.
