# Supplementary material for: How neurons exploit fractal geometry to optimize their network connectivity
Source: Sci Rep. 2021 Jan 27;11:2332. doi: 10.1038/s41598-021-81421-2 (PMC7840685; doi:10.1038/s41598-021-81421-2)
Supplement: Supplementary file 1 — Supplementary Figures. [file 41598_2021_81421_MOESM1_ESM.docx]

**Supplementary Material for “How Neurons Exploit Fractal Geometry to Optimize their Network Connectivity”**

**J.H. Smith^1,5^, C. Rowland^1,5^, B. Harland^2^, S. Moslehi^1^, R.D. Montgomery^1^, K. Schobert^1^, W.J. Watterson^1^, J. Dalrymple-Alford^3,4^, & R.P. Taylor^1,*^**

^1^Physics Department, University of Oregon, Eugene, OR 97403, USA. ^2^School of Pharmacy, University of Auckland, Auckland 1142, New Zealand. ^3^School of Psychology, Speech and Hearing, University of Canterbury, Christchurch 8041, New Zealand. ^4^New Zealand Brain Research Institute, Christchurch 8011, New Zealand. ^5^These authors contributed equally: Julian Smith, Conor Rowland. *email: [rpt@uoregon.edu](mailto:rpt@uoregon.edu)


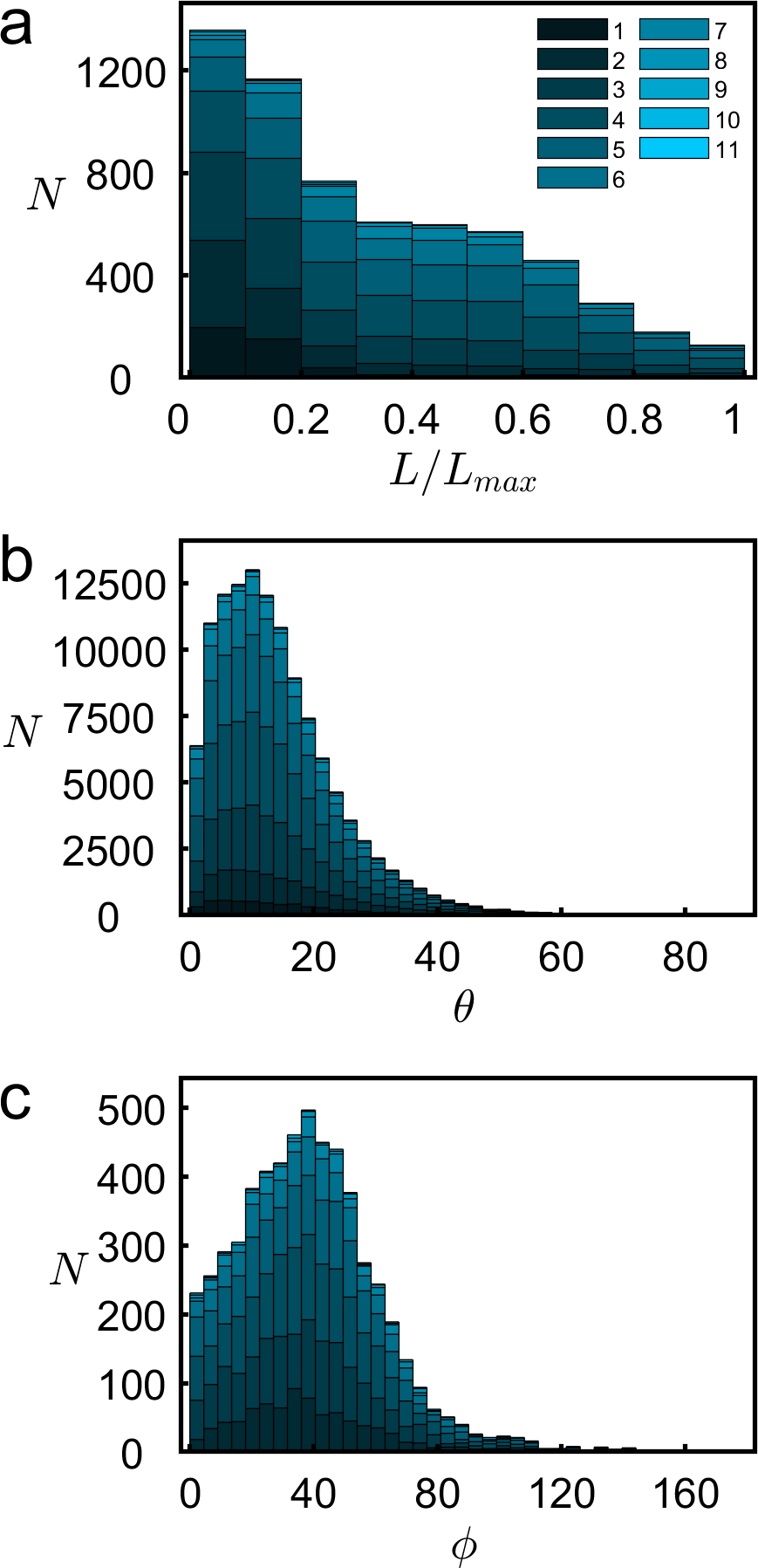


**Supplementary Figure 1:** **Length, weave angle, and fork angle distributions across all basal arbors.** (a) Histogram of the number of branches (*N*) with a normalized length (*L/L_max_*) for each branch level *i*. The legend in the upper-right corner of (a) applies to all panels in this figure and labels each branch level with a specific shade of blue. (b) Histogram of weave angles (*θ*) for each branch level *i*. (c) Histogram of fork angles (*φ*) for each branch level *i*. The 1^st^ branch level corresponds to dendrites that emerge from a neuron’s soma. As such, they do not have a fork angle. This is reflected in (c) which shows no counts for branch level 1. In each case, the histogram columns for each level are stacked on top of each other. The histogram in (a) shows that the lower branch levels are consistently shorter than the higher levels. The histograms in (b, c) don’t reveal any significant trends in terms of differences between levels.


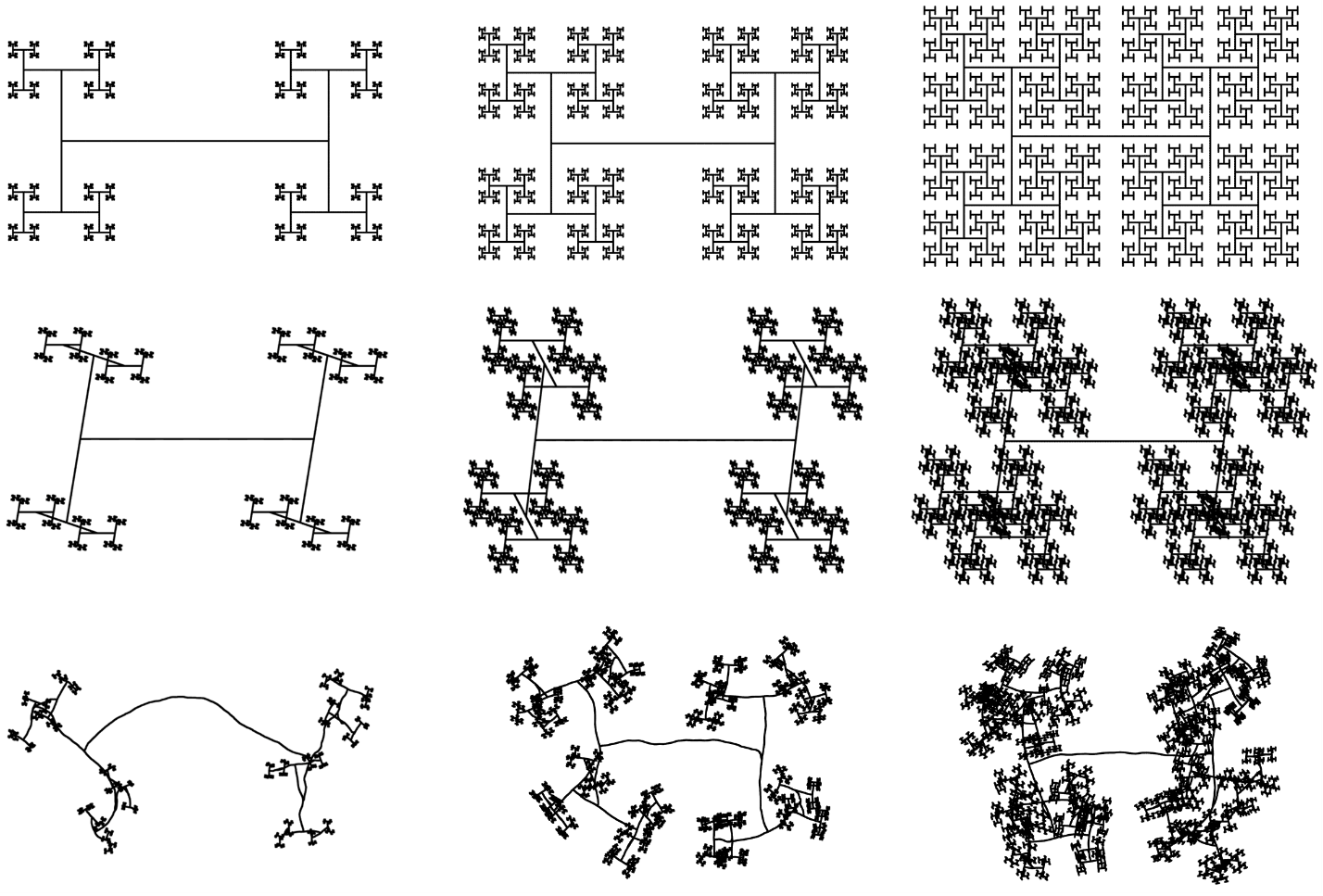


**Supplementary Figure 2: Example H-Tree models in 2D and 3D.** A visual comparison of H-Tree models (generated using Mathematica and displayed using MATLAB) extending into two-dimensional (top row) and three-dimensional (middle row) spaces for *D* = 1.1 (left), *D* = 1.5 (middle) and *D* = 1.9 (right). Their branches are straight, and their *D* values are set by the scaling relationship between branch lengths *L* at subsequent levels *i*. For the bottom row of H-Trees, the branches have been modified to introduce a distribution of weave angles. This introduction of the weave impacts the *D* value of the H-Tree.


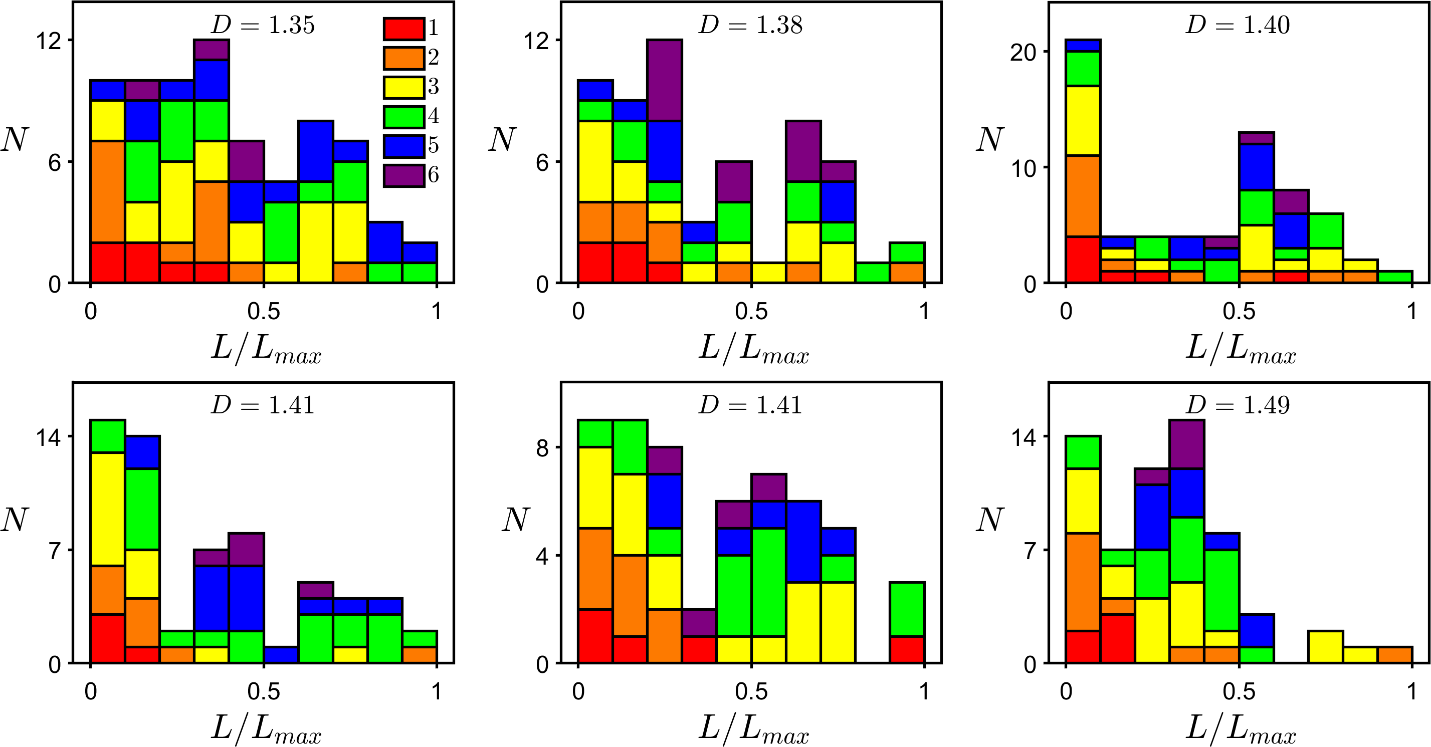


**Supplementary Figure 3: Branch distributions of six representative neurons.** Each panel shows the normalized length histogram of an individual neuron’s basal arbor for comparison with the equivalent panel of Fig. 2. The neuron *D* values are labelled in each panel. The legend shown in the top-left panel applies to all panels. The trend of lower branch levels typically featuring shorter branch lengths is observed for all six examples. There is no obvious systematic dependence of the branch length distribution on *D*.

**
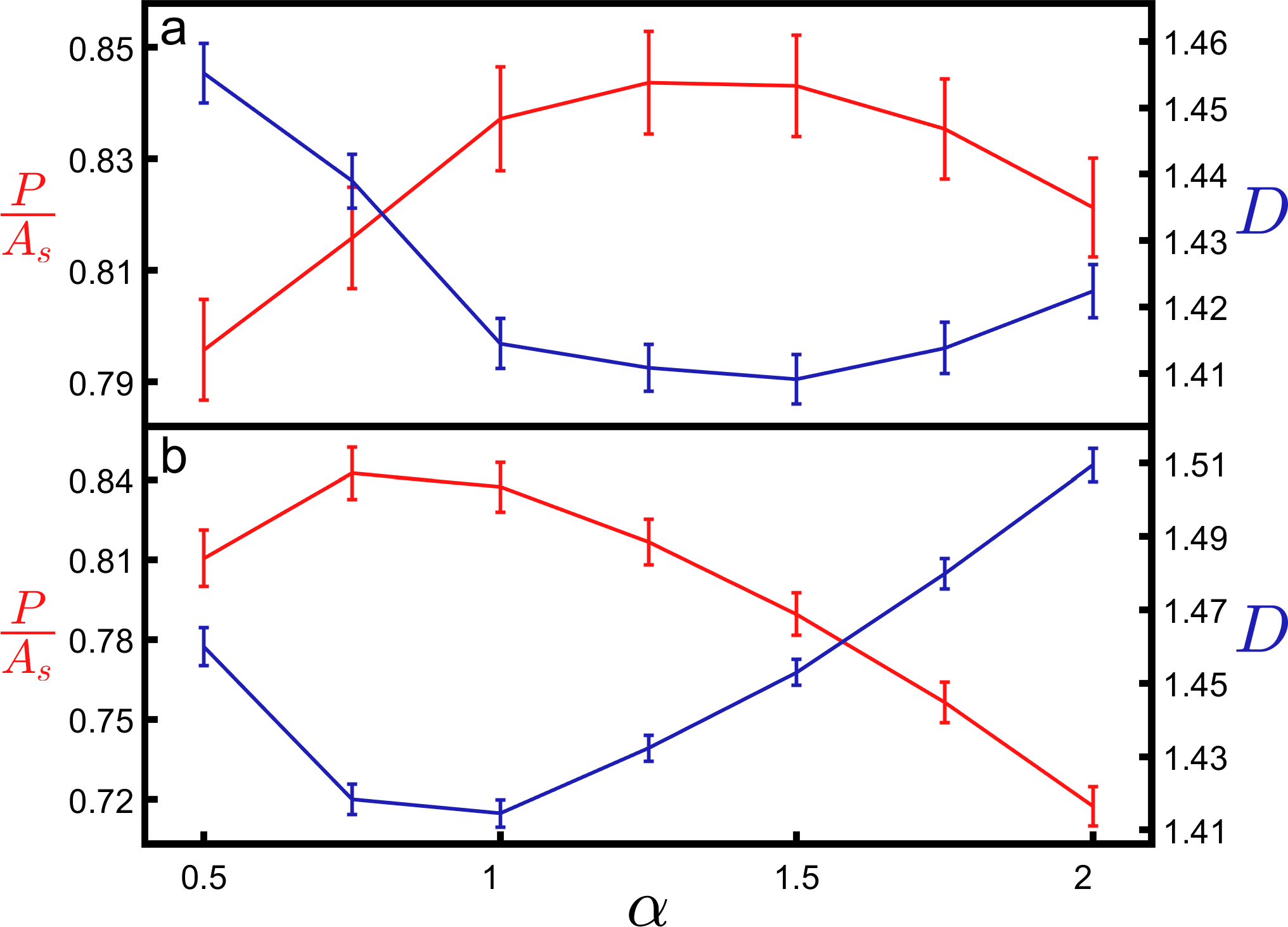
**

**Supplementary Figure 4: Effect of altering the fork angles on fractal dimension and profile.** (a) Plot of *P/A_s_* and *D* versus the angle multiplier *α* when applied only to the fork angles (*φ*). (b) An equivalent plot to (a) when *α* is applied to both *φ* and the weave angles (*θ*) simultaneously. The red and blue data are averaged over all basal arbors and their variations across arbors are represented by the shown standard errors from the mean. The plots are limited to *α* values that generate physically reasonable model conditions. Specifically, *α* values less than 0.5 are excluded to avoid very small forking angles which cause the branches to collapse into each other. As with Fig. 3, the highest value is set to be *α* = 2 to ensure that branches rarely intersect.


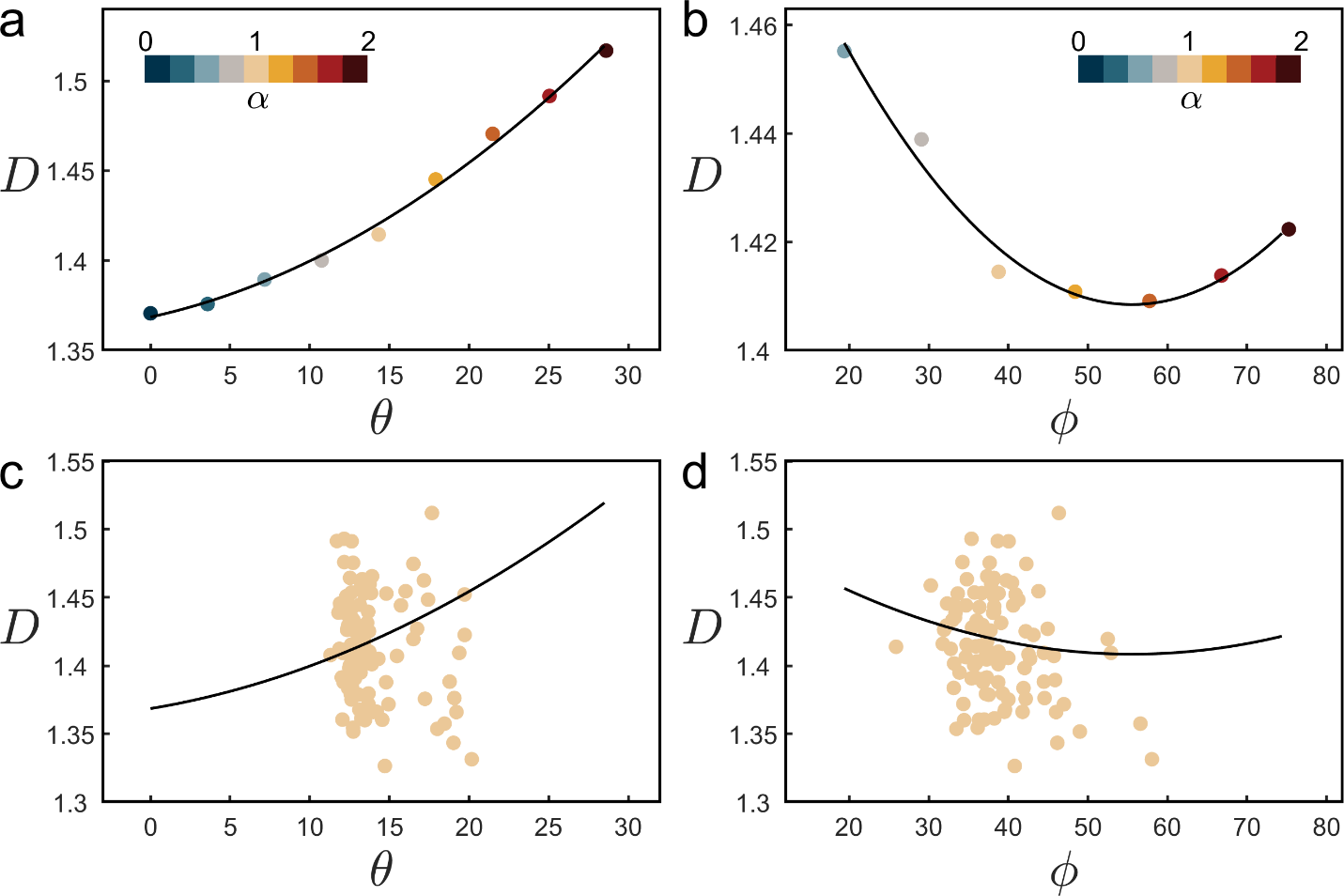


**Supplementary Figure 5: Fractal dimension dependence on the weave angles and forking angles.** (a) The mean *D* value plotted against the mean *θ* value across all basal arbors for each value of *α* used to modify *θ*. (b) The equivalent plot to (a) for *φ*. The color bars in the top-left of (a) and top-right of (b) show the colors corresponding to the various *α* values used in both (a) and (b). The black lines in (a) and (b) are 2^nd^ order polynomial fits of the data trends. (c) The *D* value of each natural basal arbor (i.e. *α* = 1) plotted against its mean *θ* value. (d) The equivalent plot to (c) for *φ*. The black lines shown in (c) and (d) correspond to those shown in (a) and (b) respectively.

**
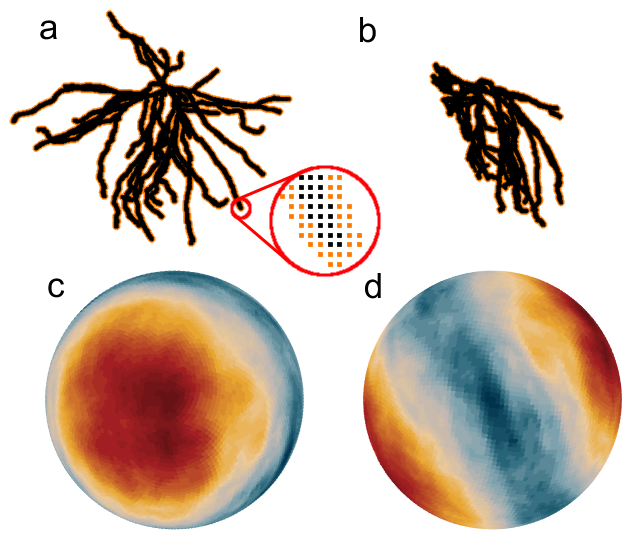
**

**Supplementary Figure 6: Measuring and mapping profile.** (a) A basal arbor (reconstructed using Neurolucida and displayed using MATLAB) viewed from the direction for which *P/A_s_* peaks. The zoom-in shows a black branch surrounded by the orange region in which spines may extend. (b) The same arbor viewed from a different direction. (c) and (d) show the equivalent profile spheres. The middle point on the sphere’s surface shown in (c) and (d) corresponds to the profile of the arbor as seen from the viewpoints shown in (a) and (b), respectively.


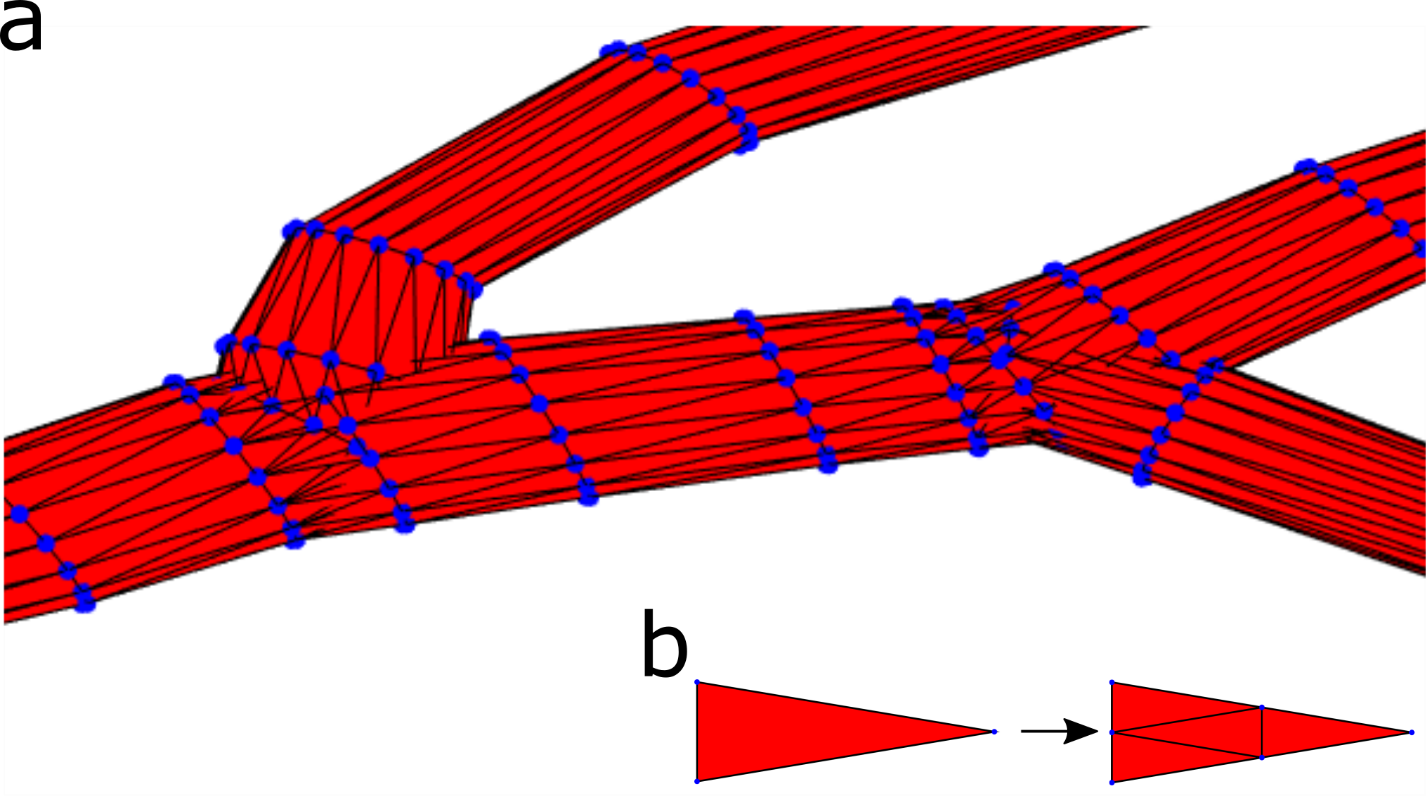


**Supplementary Figure 7: Constructing OBJ models of dendritic branches and improving surface area calculation.** (a) A close-up image of dendritic branches. The Wavefront object files (.obj) consist of cylinders constructed from vertices (blue) and triangular faces (red). (b) The triangular faces are increased 4-fold for the surface area calculations by finding the midpoints between connected vertices in a face and creating new vertices at those points. Then four new faces are created that connect the new vertices as shown.
